# Evaluation of the UroGen ELITe MGB kit for the detection of *Ureaplasma parvum*, *Ureaplasma urealyticum*, and *Mycoplasma hominis* in genital and extragenital clinical specimens

**DOI:** 10.1128/spectrum.02204-26

**Published:** 2026-09-24

**Authors:** Marie Gardette, Quentin Beaufils, Cécile Bébéar, Sabine Pereyre

**Affiliations:** 1 Bacteriology Department, National Reference Center for bacterial sexually transmitted infections, CHU de Bordeaux 36836, Bordeaux, France; 2 UMR 5234 Fundamental Microbiology and Pathogenicity, University of Bordeaux, CNRS 27086, Bordeaux, France; MultiCare Health System, Tacoma, Washington, USA; University of Louisville, Louisville, Kentucky, USA; Capital Institute of Pediatrics, Beijing, China

**Keywords:** multiplex PCR, *Mycoplasma hominis*, *Ureaplasma parvum*, *Ureaplasma urealyticum*

## LETTER

*Mycoplasma hominis*, *Ureaplasma parvum*, and *Ureaplasma urealyticum* are clinically significant human genital mycoplasmas associated with urogenital and perinatal infections (1–3). Beyond their urogenital role, these pathogens are increasingly recognized as causative agents of extra-genital infections in immunocompromised patients, in whom they have been implicated in severe conditions, such as bacteremia, septic arthritis, wound infections, and hyperammonemia, following transplantation (4–7). Diagnosis is often delayed because of the fastidious nature of these organisms and a lack of clinical awareness, underscoring the need for sensitive molecular diagnostic tools applicable across a broad range of clinical specimens (8). However, to date, no commercial kits specifically targeting these three microorganisms have been evaluated. We, therefore, evaluated the UroGen ELITe MGB multiplex PCR assay (ELITechGroup, Italy) for detection of these microorganisms in a wide range of specimen types and compared its analytical performance with previously published in-house TaqMan real-time PCR assays (9, 10).

In total, 270 clinical samples from Bordeaux University Hospital were analyzed: 141 confirmed positive (June 2019–September 2025, Table S1) and 129 negative (March 2024–April 2025). Of the positive samples, 110 involved one targeted species; 26 involved two; and five involved all three. The specimen set comprised 153 urogenital samples (vaginal, urethral, and cervical swabs; semen; urine; placental tissue; and upper genital biopsies) and 117 extra-genital samples (respiratory aspirates; ear, nose, and throat samples; cerebrospinal, joint, pleural, ascitic, pericardial, and periaortic prosthetic fluids; vascular biopsies; hip biopsy pseudomembranes; deep abscesses; blood cultures; EDTA whole blood; and spinal osteosynthesis samples). The ELITe InGenius platform was used for both DNA extraction using the ELITe InGenius SP200 kit (allowing use across a wide diversity of matrices), and target amplification: the *gap* gene for *M. hominis* and the *ureC* gene for *U. parvum* and *U. urealyticum*. In-house TaqMan real-time PCR assays were carried out using 5 µL of DNA extracts obtained from the ELITe InGenius System (9, 10). Two samples with discordant results were further resolved by 16S rRNA gene amplification and sequencing to determine the final sample status (11).

Three samples (1.1%) produced invalid results: two blood cultures and one EDTA whole-blood sample, potentially due to a blood matrix effect, as blood is known to contain PCR inhibitors (12). The positive percent agreement was 98.0, 98.9, and 100.0% for *M. hominis*, *U. parvum*, and *U. urealyticum*, respectively, whereas the negative percent agreement was 100% for all targets (Table 1). Overall, only two false-negative results were identified after discrepancy resolution, each in specimens containing two targeted species (Table S1). This could be related to competitive inhibition between co-infecting targets during amplification. However, among the 31 samples positive for more than one target, 29 were correctly detected, yielding a sensitivity of 93.5% (95% confidence interval [CI], 78.6–98.9%).

**TABLE 1:** Analytical performance of the ELITe kit^*a*^

Species	Total valid samples	In-house PCR-positive		In-house PCR-negative		Overall % agreement (95% CI)	Positive % agreement (95% CI)	Negative % agreement (95% CI)	Kappa
		ELITe-positive	ELITe-negative	ELITe-positive	ELITe-negative				
Uu	267	33	0	0	234	100 (98.6–100)	100 (89.6–100)	100 (98.4–100)	1
Up	267	92	1^*b*^	0	174	99.6 (97.9–99.9)	98.9 (94.2–99.8)	100 (97.8–100)	0.99
Mh	267	49	1^*b*^	0	217	99.3 (97.9–99.9)	98.0 (89.5–99.7)	100 (98.3–100)	0.99

^*a*^ PCR, polymerase chain reaction; CI, confidence interval; Uu,* U. urealyticum*; Up,* U. parvum*; Mh, *M. hominis*.

^*b*^ Samples confirmed positive by 16S rRNA amplification and Sanger sequencing.

Overall, the UroGen ELITe MGB assay demonstrated excellent sensitivity, specificity, and robustness across a wide range of clinical specimen types, including those containing multiple *Mycoplasma* or *Ureaplasma* species. These findings support its usefulness for routine diagnosis, particularly in upper genital tract and extra-genital infections, where rapid and reliable pathogen identification is crucial for optimal patient management.

## Supplementary Material

Reviewer comments

## SUPPLEMENTAL MATERIAL

Table S1 spectrum.02204-26-s0001.xlsx Specimen types of the 141-positive clinical samples and species identified using in-house TaqMan real-time PCR assays.

OPEN PEER REVIEW reviewer-comments.pdf An accounting of the reviewer comments and feedback.

ASM does not own the copyrights to Supplemental Material that may be linked to, or accessed through, an article. The authors have granted ASM a non-exclusive, world-wide license to publish the Supplemental Material files. Please contact the corresponding author directly for reuse.
