# Supplementary material for: Evaluation of the UroGen ELITe MGB kit for the detection of Ureaplasma parvum, Ureaplasma urealyticum, and Mycoplasma hominis in genital and extragenital clinical specimens
Source: Microbiol Spectr. 2026 Sep 24;14(11):e02204-26. doi: 10.1128/spectrum.02204-26 (PMC13729204; doi:10.1128/spectrum.02204-26)
Supplement: Supplementary file 1 [file reviewer-comments.pdf]

ASM | Microbiology Spectrum

Evaluation of the UroGen ELITe MGB kit for the detection of Ureaplasma parvum, Ureaplasma urealyticum, and Mycoplasma hominis in genital and extragenital clinical specimens

Marie Gardette, Quentin Beaufils, Cécile Bébéar, and Sabine Pereyre

Corresponding Author(s): Sabine Pereyre, Univ. Bordeaux, CNRS, UMR 5234 MFP, ARMYNE team

Review Timeline:

Submission Date:	June 26, 2026
Editorial Decision:	August 3, 2026
Revision Received:	August 18, 2026
Accepted:	September 2, 2026

Editor: Siu-Kei Chow

Reviewer(s): Disclosure of reviewer identity is with reference to reviewer comments included in decision letter(s). The following individuals involved in review of your submission have agreed to reveal their identity: Leslie A Wolf (Reviewer #1); Xue Guanhua (Reviewer #2)

Transaction Report:

(Note: With the exception of the correction of typographical or spelling errors that could be a source of ambiguity, letters and reports are not edited. The original formatting of letters and referee reports may not be reflected in this compilation.)

DOI: https://doi.org/10.1128/spectrum.02204-26

Re: Spectrum02204-26 (Evaluation of the UroGen ELITe MGB kit for the detection of Ureaplasma parvum, Ureaplasma urealyticum, and Mycoplasma hominis in genital and extragenital clinical specimens)

Dear Prof. Sabine Pereyre:

Thank you for the privilege of reviewing your work. Below you will find my comments, instructions from the Spectrum editorial office, and the reviewer comments.

Please return the manuscript within 60 days; if you cannot complete the modification within this time period, please contact me. If you do not wish to modify the manuscript and prefer to submit it to another journal, notify me immediately so that the manuscript may be formally withdrawn from consideration by Spectrum.

Revision Guidelines
To submit your modified manuscript, log into the submission site at https://spectrum.msubmit.net/cgi-bin/main.plex. Go to Author Tasks and click the appropriate manuscript title to begin. The information you entered when you first submitted the paper will be displayed; update this as necessary. Note the following requirements:

• Upload point-by-point responses to the issues raised by the reviewers in a file named "Response to Reviewers," NOT in your cover letter.
• Upload a compare copy of the manuscript (without figures) as a "Marked-Up Manuscript" file.
• Upload a clean .DOC/.DOCX version of the revised manuscript and remove the previous version.
• Each figure must be uploaded as a separate, editable, high-resolution file (TIFF or EPS preferred), and any multipanel figures must be assembled into one file.
• Any supplemental material intended for posting by ASM should be uploaded with their legends separate from the main manuscript. You can combine all supplemental material into one file (preferred) or split it into a maximum of 10 files with all associated legends included.

For complete guidelines on revision requirements, see our Submission and Review Process webpage. Submission of a paper that does not conform to guidelines may delay acceptance of your manuscript.

Data availability: ASM policy requires that data be available to the public upon online posting of the article, so please verify all links to sequence records, if present, and make sure that each number retrieves the full record of the data. If a new accession number is not linked or a link is broken, provide Spectrum production staff with the correct URL for the record. If the accession numbers for new data are not publicly accessible before the expected online posting of the article, publication may be delayed; please contact production staff (Spectrum@asmusa.org) immediately with the expected release date.

Publication Fees: For information on publication fees and which article types are subject to charges, visit our website. If your manuscript is accepted for publication and any fees apply, you will be contacted separately about payment during the production process; please follow the instructions in that e-mail. Arrangements for payment must be made before your article is published.

ASM Membership: Corresponding authors may join or renew ASM membership to obtain discounts on publication fees. Need to upgrade your membership level? Please contact Customer Service at Service@asmusa.org.

The ASM Journals program strives for constant improvement in our submission and publication process. Please tell us how we can improve your experience by taking this quick Author Survey.

Thank you for submitting your paper to Spectrum.

Sincerely,
Siu-Kei Chow
Editor
Microbiology Spectrum

Reviewer #1 (Comments for the Author):

This New Data-Letter is a concise, well-written submission regarding the comparison of the ELITe MGB kit for detection of three target organisms in genital and extra-genital clinical specimens. The authors used previously tested clinical specimens to evaluate the performance of the kit. The specimens included a wide variety of extra-genital specimens (117) in addition to more traditional urogenital specimens (153). The data presented in Table 1 were easy to interpret and appropriate methodologies were used to obtain the data.

I understand this is meant to be a short letter, but because the results are so interesting:

- I would like to see mention of in silico analysis of the primers and probes used to ensure analytical specificity.
- Some explanation of the invalid results with two blood cultures and one EDTA whole blood specimens would be helpful as well.
- Finally, I would like to see men on of additional method validation data that would be required so that this kit could be used as a lab-developed test for patient diagnosis in the US.

Reviewer #2 (Comments for the Author):

This study evaluates the UroGen ELITe MGB kit for detecting U. parvum, U. urealyticum, and M. hominis across a broad range of clinical specimens. Compared the results with in house PCR, and verified with 16s sequencing. The manuscript is well-written, and the data support the kit's utility in routine diagnostics. There are still some question need to be clarified.

1.Please add a supplemental table detailing the types and distribution of all 141 positive clinical specimens, along with the pathogen(s) detected in each specimen. This table should indicate, for each positive sample, the specimen type (e.g., blood culture, joint fluid, respiratory aspirate, cerebrospinal fluid, urogenital swab, etc.) and the specific target(s) identified (M. hominis, U. parvum, and/or U. urealyticum), clearly distinguishing mono-infections from co-infections. This would allow readers to assess the kit's performance across different matrices and better support the authors' claim of broad specimen applicability.

2.The two false negatives occurred in co-infected samples, which worth discussing whether this reflects competitive inhibition or target-specific lower sensitivity.

Manuscript: Spectrum02204-26

"Evaluation of the UroGen ELITe MGB kit for the detection of *Ureaplasma parvum*, *Ureaplasma urealyticum*, and *Mycoplasma hominis* in genital and extragenital clinical specimens", Corresponding Author: Sabine Pereyre

Reviewer Comments for New Data-Letter

This New Data-Letter is a concise, well-written submission regarding the comparison of the ELITe MGB kit for detection of three target organisms in genital and extra-genital clinical specimens.

The authors used previously tested clinical specimens to evaluate the performance of the kit. The specimens included a wide variety of extra-genital specimens (117) in addition to more traditional urogenital specimens (153). The data presented in Table 1 were easy to interpret and appropriate methodologies were used to obtain the data.

I understand this is meant to be a short letter, but because the results are so interesting, I would like to see mention of *in silico* analysis of the primers and probes used to ensure analytical specificity. Some explanation of the invalid results with two blood cultures and one EDTA whole blood specimens would be helpful as well. Finally, I would like to see mention of additional method validation data that would be required so that this kit could be used as a lab-developed test for patient diagnosis in the US.

Dear Dr Siu-Kei Chow
Please find our point-by-point responses to the reviewer comments.
Best regards
Sabine Pereyre

Reviewer #1 (Comments for the Author):

This New Data-Letter is a concise, well-written submission regarding the comparison of the ELITe MGB kit for detection of three target organisms in genital and extra-genital clinical specimens. The authors used previously tested clinical specimens to evaluate the performance of the kit. The specimens included a wide variety of extra-genital specimens (117) in addition to more traditional urogenital specimens (153). The data presented in Table 1 were easy to interpret and appropriate methodologies were used to obtain the data.

I understand this is meant to be a short letter, but because the results are so interesting:

- I would like to see mention of in silico analysis of the primers and probes used to ensure analytical specificity.

We agree that an in silico analysis of the primers and probes would be relevant to further assess analytical specificity. However, as this is a commercially available kit, the sequences of the primers and probes are not disclosed by the manufacturer. The instructions for use (IFU) provided by the manufacturer report the analytical specificity of the kit. Notably, no cross-reactivity or interference was observed with the panel of bacteria, viruses, and yeasts tested, as listed in the IFU.

- Some explanation of the invalid results with two blood cultures and one EDTA whole blood specimens would be helpful as well.

We agree with the reviewer that an explanation for the invalid results observed in blood specimens would be helpful. Indeed, three samples (1.1%) produced invalid results: two blood cultures and one EDTA whole blood sample. These invalid results could potentially be explained by a blood matrix effect, as blood is known to contain PCR inhibitors, mainly hemoglobin and immunoglobulin G, which can interfere with DNA polymerase activity and reduce amplification efficiency. This explanation has been added to the revised manuscript (lines 57-58), together with a reference supporting this point (Sidstedt et al. 2018).

- Finally, I would like to see mention of additional method validation data that would be required so that this kit could be used as a lab-developed test for patient diagnosis in the US.

We agree that additional analytical and clinical validation would be required if the assay were to be implemented as a laboratory-developed test (LDT) for patient diagnosis in the United States. However, our study focused on the evaluation of the commercially available assay according to its intended use. The analytical performance characteristics of the assay are provided in the manufacturer's instructions for use (IFU). Given the strict word limit of this New Data Letter, we were unable to provide further details on LDT-specific validation requirements.

Reviewer #2 (Comments for the Author)

This study evaluates the UroGen ELITe MGB kit for detecting U. parvum, U. urealyticum, and

M. hominis across a broad range of clinical specimens. Compared the results with in house PCR, and verified with 16s sequencing. The manuscript is well-written, and the data support the kit's utility in routine diagnostics. There are still some question need to be clarified.

1.Please add a supplemental table detailing the types and distribution of all 141 positive clinical specimens, along with the pathogen(s) detected in each specimen. This table should indicate, for each positive sample, the specimen type (e.g., blood culture, joint fluid, respiratory aspirate, cerebrospinal fluid, urogenital swab, etc.) and the specific target(s) identified (M. hominis, U. parvum, and/or U. urealyticum), clearly distinguishing mono-infections from co-infections. This would allow readers to assess the kit's performance across different matrices and better support the authors' claim of broad specimen applicability.

This is a very good suggestion, as it allows readers to better assess the kit's performance across different specimen matrices and further supports our claim of broad specimen applicability. As suggested, we added the requested table as supplemental Table S1, detailing the specimen types of all 141 positive clinical specimens, along with the specific target(s) identified for each sample.

2.The two false negatives occurred in co-infected samples, which worth discussing whether this reflects competitive inhibition or target-specific lower sensitivity.

We agree with the reviewer that this point deserves further discussion. Indeed, the two false-negative results occurred in co-infected samples. We do not think that target-specific lower sensitivity explains these results, as *M. hominis* was not detected in one case, whereas *U. parvum* was not detected in the other (Table S1). These two false-negative results could therefore be related to competitive inhibition between co-infecting targets during amplification. However, as mentioned in lines 62–64 of the original manuscript, among the 31 samples positive for more than one target, 29 were correctly detected, corresponding to a sensitivity of 93.5%. This point has been further specified in lines 63–66 of the revised manuscript.

Re: Spectrum02204-26R1 (Evaluation of the UroGen ELITe MGB kit for the detection of Ureaplasma parvum, Ureaplasma urealyticum, and Mycoplasma hominis in genital and extragenital clinical specimens)

Dear Prof. Sabine Pereyre:

Your manuscript has been accepted, and I am forwarding it to the ASM production staff for publication. Your paper will first be checked to make sure all elements meet the technical requirements. ASM staff will contact you if anything needs to be revised before copyediting and production can begin. Otherwise, you will be notified when your proofs are ready to be viewed.

Data Availability: ASM policy requires that data be available to the public upon online posting of the article, so please verify all links to sequence records, if present, and make sure that each number retrieves the full record of the data. If a new accession number is not linked or a link is broken, provide production staff with the correct URL for the record. If the accession numbers for new data are not publicly accessible before the expected online posting of the article, publication may be delayed; please contact ASM production staff immediately with the expected release date.

Publication Fees: For information on publication fees and which article types have charges, please visit our website. We have partnered with Copyright Clearance Center (CCC) to collect author charges. If fees apply to your paper, you will receive a message from no-reply@copyright.com with further instructions. For questions related to paying charges through RightsLink, please contact CCC at ASM_Support@copyright.com or toll free at +1-877-622-5543. CCC makes every attempt to respond to all emails within 24 hours.

ASM Membership: Corresponding authors may join or renew ASM membership to obtain discounts on publication fees. Need to upgrade your membership level? Please contact Customer Service at Service@asmusa.org.

Embargo Policy: A press release may be issued as soon as the manuscript is posted on the Spectrum Latest Articles webpage. The corresponding author will receive an email with the subject line "ASM Journals Author Services Notification" when the article is available online.

Cover Image Submissions: If you would like to submit a potential Cover Image, please email a file and a short legend to spectrum@asmusa.org. Please note that we can only consider images that (i) the authors created or own and (ii) have not been previously published. By submitting, you agree that the image can be used under the same terms as the published article. Image File requirements: TIF/EPS, 7.5 inches wide by 8.25 inches tall (at least 2,250 pixels wide by 2,475 pixels tall), minimum 300 dpi resolution (600 dpi preferred), RGB, and no figure elements, e.g., arrows or panel labels. The legend should be a short description of the image, 1-2 sentences recommended. Please use the ASM Journals Cover Image Sizing Tool (https://journals.asm.org/pb-assets/html/asm-cover-sizing-tool.html) to ensure that your proposed cover image meets our size requirements.

The ASM Journals program strives for constant improvement in our submission and publication process. Please tell us how we can improve your experience by taking this quick Author Survey.

Thank you for submitting your paper to Spectrum.

Sincerely,
Siu-Kei Chow
Editor
Microbiology Spectrum
